# The rejuvenating effect of pregnancy on muscle regeneration

**DOI:** 10.1111/acel.12286

**Published:** 2015-03-13

**Authors:** Tal Falick Michaeli, Neri Laufer, Jitka Yehudit Sagiv, Avigail Dreazen, Zvi Granot, Eli Pikarsky, Yehudit Bergman, Yuval Gielchinsky

**Affiliations:** 1 Rubin Chair in Medical Science, Department of Developmental Biology & Cancer Research, IMRIC, Hebrew University-Hadassah Medical School91120, Jerusalem, Israel; 2 Department of Obstetrics & Gynecology, Hadassah-Hebrew University Medical Center91120, Jerusalem, Israel; 3 Department of Biochemistry & Molecular Biology, IMRIC, Hebrew University-Hadassah Medical School91120, Jerusalem, Israel; 4 Department of Pathology & the Lautenberg Center for Immunology, IMRIC, Hebrew University-Hadassah Medical School91120, Jerusalem, Israel

**Keywords:** aging, muscle, pregnancy, regeneration, rejuvenation

## Abstract

Aging is characterized by reduced tissue regenerative capacity attributed to a diminished responsiveness of tissue-specific stem cells. With increasing age, resident precursor cells in muscle tissues show a markedly impaired propensity to proliferate in response to damage. However, exposure to factors present in the serum of young mice restores the regenerative capacity of aged precursor cells. As pregnancy represents a unique biological model of a partially shared blood system between young and old organisms, we hypothesized that pregnancy in aged mice would have a rejuvenating effect on the mother. To test this hypothesis, we assessed muscle regeneration in response to injury in young and aged pregnant and nonpregnant mice. Muscle regeneration in the aged pregnant mice was improved relative to that in age-matched nonpregnant mice. The beneficial effect of pregnancy was transient, lasting up to 2 months after delivery, and appeared to be attributable to activation of satellite cells via the Notch signaling pathway, thus supporting the possibility that pregnancy induces activation of aged dormant muscle progenitor cells.

Aging is characterized by a decline in regenerative capacity, which can be partially explained by changes in growth factors, accumulation of DNA damage, and lowered responsiveness of progenitor cells. Adult mammalian skeletal muscle is a stable tissue, yet is able to undergo extensive regeneration in response to damage. Muscle progenitor cells, termed satellite cells, give rise to proliferative myoblasts that fuse to form myotubes (Chargé & Rudnicki, 2004).

A considerable body of research focused on negative effects of aging on skeletal muscle regeneration. The activation of aged satellite cells and the regenerative potential of aged muscle can be restored by forced activation of Notch signaling pathway, demonstrating that the intrinsic regenerative capacity of aged satellite cells remains intact (Conboy *et al*., 2003). In rats, aged muscle successfully regenerates when grafted into a young host, and young muscle displays impaired regeneration when grafted into an aged host. Heterochronic parabiosis in mice (connecting the blood circulations of a young and an old animal) can restore muscle regenerative capacity (Conboy *et al*., 2005). Pregnancy can be viewed as a natural state akin to parabiosis, where organisms partly share blood systems – in this case, an adult organism (the pregnant mother) is exposed to extremely young organisms (the fetuses). We recently showed that pregnancy restores the regenerative capacity of the aged liver in mice (Gielchinsky *et al*., 2010). We therefore set out to examine whether pregnancy affects the declining capacity for muscle regeneration in old mice.

First, we examined the regenerative capacity of mouse muscle tissue in young (2–3 months), aged (10 months), and old females (>18 months), by immunostaining injured muscle for eMHC, a marker of regenerating myotubes. Five days after injury, muscles in young mice showed robust regeneration, with a mean regeneration index (RI) of 26 ± 3%, obtained by expressing the eMHC-stained area as a percentage of the injured area. In contrast, injured muscle from old mice regenerated poorly (RI = 2.8%). Regenerative efficacy in aged mice (RI = 21 ± 3%) did not differ significantly from that of the young mice. Interestingly, muscle regeneration during pregnancy both in young (RI = 57 ± 17%) and in aged (RI = 45 ± 7%) mice was significantly improved relative to nonpregnant mice (*P *< 0.01 and *P *< 0.01, respectively, Student’s *t*-test, Fig.1A,B and Fig. S1a,b). Therefore, pregnancy enhances muscle regeneration in young and aged mice.

**Fig 1:**
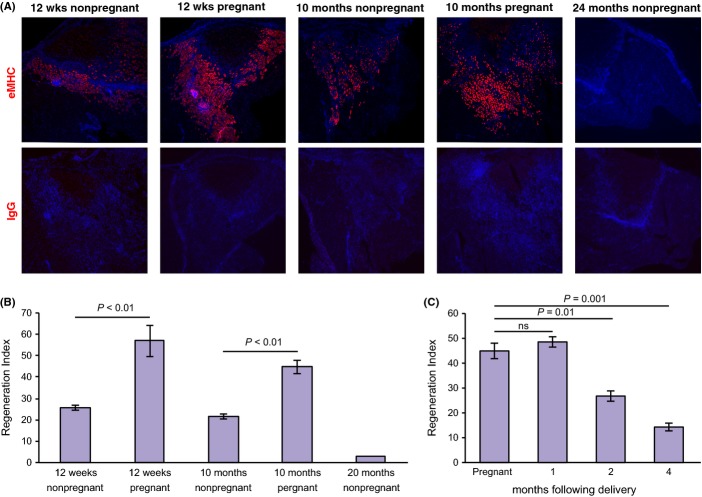
Pregnancy enhances muscle regeneration. (A) eMHC immunofluorescence staining, 5 days after injury. Upper panel: eMHC (red); Dapi (blue). Lower panel IgG control (B) Quantification of experiments in A. (mean ± SEM; *n *= 5). (C) Quantification of regenerative response at indicated time points after delivery (mean ± SEM; *n *= 5).

To study the duration of the beneficial effect of pregnancy, we examined muscle regenerative efficacy in aged mice at several time points after delivery. For up to 1 month after delivery, RI was similar to that in aged pregnant mice (49 ± 4%; Fig.1C). Enhanced RI was not observed later on (27 ± 4% at 2 months and 14 ± 3% at 4 months after delivery); hence, the beneficial effect of pregnancy, although lasting for several weeks, is temporary.

Can pregnancy improve regeneration in old mice? As old mice cannot conceive, we induced parabiosis between young pregnant mice and old nonpregnant mice. As a control, parabiosis was set up between young nonpregnant and old nonpregnant mice. Remarkably, parabiosis with young pregnant mice enhanced muscle regeneration in the old partners significantly more than with young nonpregnant mice (RI = 57 ± 12% vs. RI = 20 ± 8%, *P *= 0.05, Mann–Whitney one-tailed *U*-test; Fig.2A and Fig. S2a). These results indicated that pregnancy enhances muscle regeneration in old mice as well, and suggested that the impaired regenerative potential of old satellite cells can be improved by modification of the systemic environment via an increase in factors from pregnant mouse serum.

**Fig 2:**
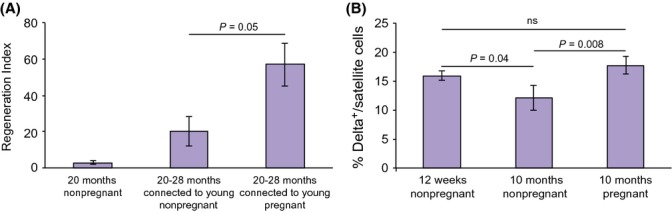
Pregnancy enhances muscle regeneration in old mice. (A) Quantification of regeneration index (RI) in old parabiotic partner (20–28 month), 5 days after injury (*n *= 3). RI in nonparabiosed old mice (20 month) is shown for comparison. (B) Satellite cell activation after muscle injury in young, aged, and aged pregnant mice.

To determine whether the observed beneficial effect was due to activation of resident progenitor cells or engraftment of cells from the embryos, regenerating muscle tissue was examined 5 days after injury in pregnant mice carrying fetuses harboring an EGFP transgene. Although GFP-positive embryos were present, no EGFP-positive cells were detectable at the site of injury (Fig. S2b,c), suggesting that microchimerism is not a major mechanism for restoring regenerative potential in aged mothers.

To determine whether the source of the circulating rejuvenating factor was maternal (e.g., ovary, decidua) or fetal, we tested the effect of pseudopregnancy (a state in which transient alteration of maternal pituitary and ovarian hormones mimics the changes during the first half of normal gestation) on muscle regeneration. RI in the pseudopregnant mice (39 ± 5%) was significantly higher than in the nonpregnant state (21 ± 3%; *P* < 0.01) and was close to that of pregnant mice (45 ± 7%). These findings suggested that a significant part of enhanced regeneration in pregnant mice can be attributed to maternally derived factors.

As progesterone is a major pregnancy hormone increasing during pregnancy and in pseudopregnancy, we tested the effect of administering progesterone via osmotic pumps on muscle regeneration. There was no significant difference in RIs between aged mice receiving progesterone to those receiving vehicle alone.

Next, we wanted to determine whether the beneficial effect of pregnancy on muscle regeneration, demonstrated by regenerating myotubes, is due to an increase in satellite cell number. We quantified purified satellite cells isolated from mouse hindlimb muscles in young, aged, and aged pregnant mice. Satellite cells (VCAM1^+^/CD31^−^/CD45^−^/Sca1^−^) were isolated using FACS, 36 h after injury. No significant difference was found between the groups. Therefore, the higher regeneration capacity is not attributed to an increased number of satellite cells.

Age-related decrease in muscle regenerative potential was found to be due to a decline in Notch signaling and can be reversed by Notch activation, as measured by upregulation of Delta-1 expression in satellite cells (Conboy *et al*., 2003). Thus, we compared Delta expression in satellite cells isolated from injured mouse muscles in young, aged, and aged pregnant mice. Notably, we found a higher percentage of activated satellite cells expressing the Delta ligand in aged pregnant mice, as compared to aged mice. In contrast, similar Delta expression levels were observed in young and aged pregnant mice, indicating that pregnancy restored Notch activation of aged mice to the level of young mice (Fig.2B). Therefore, we concluded that pregnancy, through Notch activation, promoted the regeneration capacity of injured muscle in aged mice, suggesting that ‘pregnancy-rejuvenating’ factor can overcome the negative effect of age.

We recently demonstrated that pregnancy markedly improves liver regeneration in aged mice (Gielchinsky *et al*., 2010). Furthermore, an enhanced ability to remyelinate white matter lesions was reported in pregnant mice (Gregg *et al*., 2007). These two studies support the notion that pregnancy has a rejuvenating effect on the regenerative capacity of different maternal organs. This effect is probably not due to a single common pathway, but rather to specific mechanisms in different tissues. Identification of the maternally derived pregnancy factor(s) that enhance muscle regenerative potential could have far-reaching therapeutic implications in the aging population.

## Supporting Information

**Appendix S1.** Experimental procedures.

**Fig. S1** (a) Quantification of injury and regeneration using image analysis technology. Immunostaining for activated satellite cells with anti-eMHC antibody was performed 5 days after muscle injury. The percentage of eMHC-positive cells was assessed using an automated scanning microscope and image analysis system. The regeneration index (RI) was calculated as the area of eMHC-positive fibers at the injury site and normalized to the total area of the injury site, marked with blue line. (RI = eMHC-stained area/injured area, expressed as a percentage). (b) Pregnancy enhances muscle regeneration. Muscles were analyzed for regeneration, 5 days after injury, by immunohistochemical staining for eMHC (upper panel). Lower panel IgG control. The yellow line demarcates the necrotic area.

**Fig. S2** (a) Representative blood smear demonstrating blood chimerism in a parabiotic partner. The green cells are white blood cells from the circulation of the nontransgenic parabiotic partner, derived from the GFP-transgenic mouse. Blue, Hoechst nuclear staining. (b) Pregnancy enhances muscle regeneration in old mice, but not through engraftment of circulating progenitor cells from embryos to the mother. C57/BL females were mated with a GFP-transgenic male mouse. Using a camera with a charged-couple device (CCD), GFP-positive embryos were identified in the wild-type mothers. (c) Immunofluorescence staining with an anti-GFP antibody, 5 days after muscle injury to the pregnant mother, did not reveal GFP-positive (green) cells at the injured site. Dapi (blue) labels all nuclei. eMHC (red) (*n* = 3).
