# Supplementary material for: The rejuvenating effect of pregnancy on muscle regeneration
Source: Aging Cell. 2015 Mar 13;14(4):698–700. doi: 10.1111/acel.12286 (PMC4531083; doi:10.1111/acel.12286)
Supplement: Supplementary file 1 [file acel0014-0698-sd1.doc]

Online Supporting Information

**Experimental Procedures**

**Animal strains and animal studies**

All animal experiments were performed in accordance with IACUC guidelines. Young (2-3 month) female C57/BL mice were obtained from Harlan Laboratories, Jerusalem, Israel and young (2-3 month) GFP-transgenic mice (C57BL/6-Tg(ACTB-EGFP)1Osb/J) from Jackson Laboratories, England. Female aged (10-12 month) and old (18-24 month) mice were purchased from the National Institute of Aging, Baltimore, MD, USA and from Charles River Laboratories, France. In addition, some mice were maintained up to the required age in the Specific Pathogen-Free (SPF) animal facility at our institution (Hebrew University-Hadassah Medical School Jerusalem, Israel). All mice were housed in the SPF.

**Antibodies**

The eMyHC antibody was developed by H.M. Blau under the auspices of the National Institute of Child Health and Human Development (NICHD) and maintained by the University of Iowa, Department of Biological Sciences, Iowa City, IA 52242, USA. We obtained the antibody from the Developmental Studies Hybridoma Bank, University of Iowa. The specificity of the antibody was validated (Fig.1A, S1B). Anti-GFP antibody (A6455) was from Molecular Probes. FACS antibodies: mouse CD31-APC (Biolegend), mouse CD45-APC (Biolegend), mouse Sca1-Pacific-Blue (Biolegend), mouse VCAM1-biotin (Biolegend), Streptavidin-PE-Cy7 (Biolegend), Delta (Santa Cruz, CA). Mouse IgG1 (Cell Signaling).

**Muscle injury and muscle preparation**

Tissue response to injury:

Prior to *in-vivo* satellite cell activation and analysis of muscle regeneration, mice (under general anesthetic) were subjected to a small, reproducible “wedge” injury by application of a cold metal bar (a 5-mm spatula previously cooled in liquid nitrogen) directly to the tibialis anterior muscle for 10 seconds. The insult produced a discrete border between uninjured and injured muscle, and this remained clear and distinct during regeneration of the injured tissue. The skin incision was stitched with silk sutures. Five days later the mice were euthanized and the muscle was dissected, embedded in Tissue Tek® OCT™ Compound, and placed on a metal plate, which was put into a bath filled with Isopentane. This was placed in turn in a larger bath with liquid nitrogen. The frozen muscles were kept at -70°C.

Satellite cell activation and isolation:

In order to induce activation of satellite cells for analysis of in vivo activation by flow cytometry, hindlimb muscles (tibialis anterior, gastrocnemius, and quadriceps) were pierced multiple times with a 23-gauge needle. (Conboy IM, Conboy MJ, Smythe GM, Rando TA. (2003). Notch-mediated restoration of regenerative potential to aged muscle. Science. 302:1575-7).

Satellite cell isolation was performed as previously described (Liu L, Cheung TH, Charville GW et al (2013) Chromatin modifications as determinants of muscle stem cell quiescence and chronological aging. Cell Rep. 4:189-204). Hindlimb muscles were dissected from mice following euthanization and kept in wash solution (Ham’s F10 media supplemented with 10% horse serum). The muscles were then finely minced with a surgical scalpel and digested in 2 mg/ml Collagenase II (Worthington, 340 U/mg) in the wash solution for 90 min with agitation. Dissociated fibers were washed with wash solution and further digested in 10 ml wash solution containing 100 U/ml Collagenase II and 2 U/ml Dispase (Invitrogen) for 30 min in shaking water bath. The resulting suspension was then passed through a 20-G needle with a 10-ml syringe 10 times to release the associated satellite cells. Cell suspensions were then wash two more times and filtered through a 45-mm cell strainer. Mononuclear cells from each mouse were resuspended in 500 ml wash solution and stained with VCAM1-biotin, CD31-APC, CD45- APC and Sca1-Pacific-Blue antibodies at 1:100 dilution at 4°C with gentle agitation for 30 min. Cells were washed once and resuspended in 500 ml wash solution and stained with Streptavidin-PE-Cy7 at a 1:100 dilution at 4°C with gentle agitation for 15 min. The resulting preparation contains satellite cells (VCAM1+/CD31-/CD45-/Sca1-), which could be distinguished from remaining cells and debris using flow cytometry.

**Immunohistochemistry and immunofluorescence of frozen muscle sections**

For embryonic myosin heavy chain (eMHC) immunostaining, a specific marker of regenerating myotubes in adults, frozen sections (5 µM) were fixed immediately in cooled acetone (-20°C) and then immersed for 1 h in 4% formaldehyde buffer at room temperature. After immersion for 5 min in 3% H_2_O_2_, slides were placed in 25 mM citrate buffer, pH 6.0, and heated in a pressure cooker at 115°C for 3 min (Decloaking Chamber™), and then transferred to boiling deionized water and allowed to cool for 20 min. Slides were incubated with mouse monoclonal anti-eMHC antibodies diluted 1:100 in CAS-Block™ (Zymed Laboratories), and kept overnight at 4°C. They were then washed three times with OptiMax (HK583 Wash Buffer, BioGenex Laboratories), incubated for 30 min with anti-mouse Envision+ K4007 (DakoCytomation), and developed for 15 min with 3,3′-diaminobenzidine (DAB).

**Effectiveness of muscle regeneration**

The effectiveness of muscle regeneration was quantified by determination of the regeneration index (RI). Immunostaining for activated satellite cells with anti-eMHC antibody was performed 5 days after muscle injury. The percentage of eMHC-positive cells was assessed using an Ariol SL-50® automated scanning microscope and image analysis system (Applied Imaging, Grand Rapids, MI, USA), according to the manufacturer's instructions. The RI was calculated as the area of eMHC-positive fibers at the injury site and normalized to the total area of the injury site. The same gating parameters were used for all sections (Fig. S1A).

**Parabiotic pairing**

Parabiosis between a young female and an old female mouse was performed as previously described, with some modifications (Bunster E, Meyer RK (1933) An improved method of parabiosis. *Anat. Rec*. 75, 339-343). Both mice were anesthetized to full muscle relaxation by intraperitoneal injection with ketamine HCl and xylazine. The corresponding lateral aspects of the two partners were shaved, matching skin incisions were made from the olecranon to the knee joint in both mice, and their subcutaneous fasciae were bluntly dissected to create about 0.5 cm of free skin. An incision was made in the peritoneum of each mouse, and both peritonea were tied with 4-0 vicryl sutures. The dorsal and ventral skins were approximated by continuous silk sutures. To confirm the establishment of a shared blood system between the two parabionts, a blood smear was obtained from the wild-type female 7 days after the operation. The presence of GFP+ white blood cells in this blood smear demonstrated that blood chimerism had been established, and that the two mice now shared a common blood system (Fig. S2A). Next, the old parabiont was subjected to thermal injury of the tibialis anterior muscle. Regeneration efficacy was examined 5 days later. Parabiosis with a young pregnant partner was performed where indicated on pregnancy day 8**.**

**Engraftment of embryonal circulating progenitors to the injury site**

A GFP-transgenic male mouse was mated C57/BL females. Using a MagnaFire SP digital camera (<http://www.lightools.com/index.htm>), GFP-positive embryos were identified in these wild-type females on day 14 post-coitus. The pregnant mice were subjected to thermal injury of the tibialis anterior muscle on day 14 of the pregnancy. The muscles were harvested 5 days after the injury and frozen sections were prepared. Immunostaining was performed with anti-GFP antibody, and GFP-positive cells were identified at the injured site using a fluorescence microscope. The observed presence of GFP-positive cells in the maternal tissue supported the engraftment hypothesis (Fig. S2B, S2C).

**Pseudopregnancy**

Aged mice (10-12 months) were mated with vasectomized males. Progesterone was assayed 4 days after vaginal plugs were observed. Mice with elevated progesterone were considered to be pseudopregnant (Shiotani M, Noda Y, Mori T (1993) Embryo-dependent induction of uterine receptivity assessed by an in-vitro model of implantation in mice. *Biol. Reprod.* 49, 794-801). The female mice were subjected to muscle injury 9 days after mating.

**Implantation of the osmotic pumps and progesterone infusion**

Aged female mice (10-12 months) were anesthetized with ketamine/xylazine anesthesia and implanted with a micro-osmotic pump (Alzet 1002, Alza Corp., Palo Alto, CA). The cannula was located paramedially on the right side of the back for subcutaneous infusions. Progesterone (Sigma) was dissolved in DMSO and was infused at a rate of 0.25 mg/d. Each animal was infused with progesterone or vehicle for 12 consecutive days. On day 7 muscle injury was performed, muscles were harvested 5 days later.

**Statistics**

All values are means ± SEM. The indicated statistical tests were performed using StatXact software. Where applicable, two-tailed tests were applied.

**Supplementary data - Figure Legends**

**Fig. S1** (A) Quantification of injury and regeneration using image analysis technology. Immunostaining for activated satellite cells with anti-eMHC antibody was performed 5 days after muscle injury. The percentage of eMHC-positive cells was assessed using an automated scanning microscope and image analysis system. The regeneration index (RI) was calculated as the area of eMHC-positive fibers at the injury site and normalized to the total area of the injury site, marked with blue line. (RI = eMHC-stained area/injured area, expressed as a percentage). (B) Pregnancy enhances muscle regeneration. Muscles were analyzed for regeneration, 5 days after injury, by immunohistochemical staining for eMHC (upper panel). Lower panel IgG control. The yellow line demarcates the necrotic area.

**Fig. S2** (A) Representative blood smear demonstrating blood chimerism in a parabiotic partner. The green cells are white blood cells from the circulation of the nontransgenic parabiotic partner, derived from the GFP-transgenic mouse. Blue, Hoechst nuclear staining.

(B) Pregnancy enhances muscle regeneration in old mice, but not through engraftment of circulating progenitor cells from embryos to the mother. C57/BL females were mated with a GFP-transgenic male mouse. Using a camera with a charged-couple device (CCD), GFP-positive embryos were identified in the wild-type mothers. (C) Immunofluorescence staining with an anti-GFP antibody, 5 days after muscle injury to the pregnant mother, did not reveal GFP-positive (green) cells at the injured site. Dapi (blue) labels all nuclei. eMHC (red) (*n* = 3).
