# Supplementary material for: The rejuvenating effect of pregnancy on muscle regeneration
Source: Aging Cell. 2015 Mar 13;14(4):698–700. doi: 10.1111/acel.12286 (PMC4531083; doi:10.1111/acel.12286)
Supplement: Supplementary file 2 [file acel0014-0698-sd2.pdf]

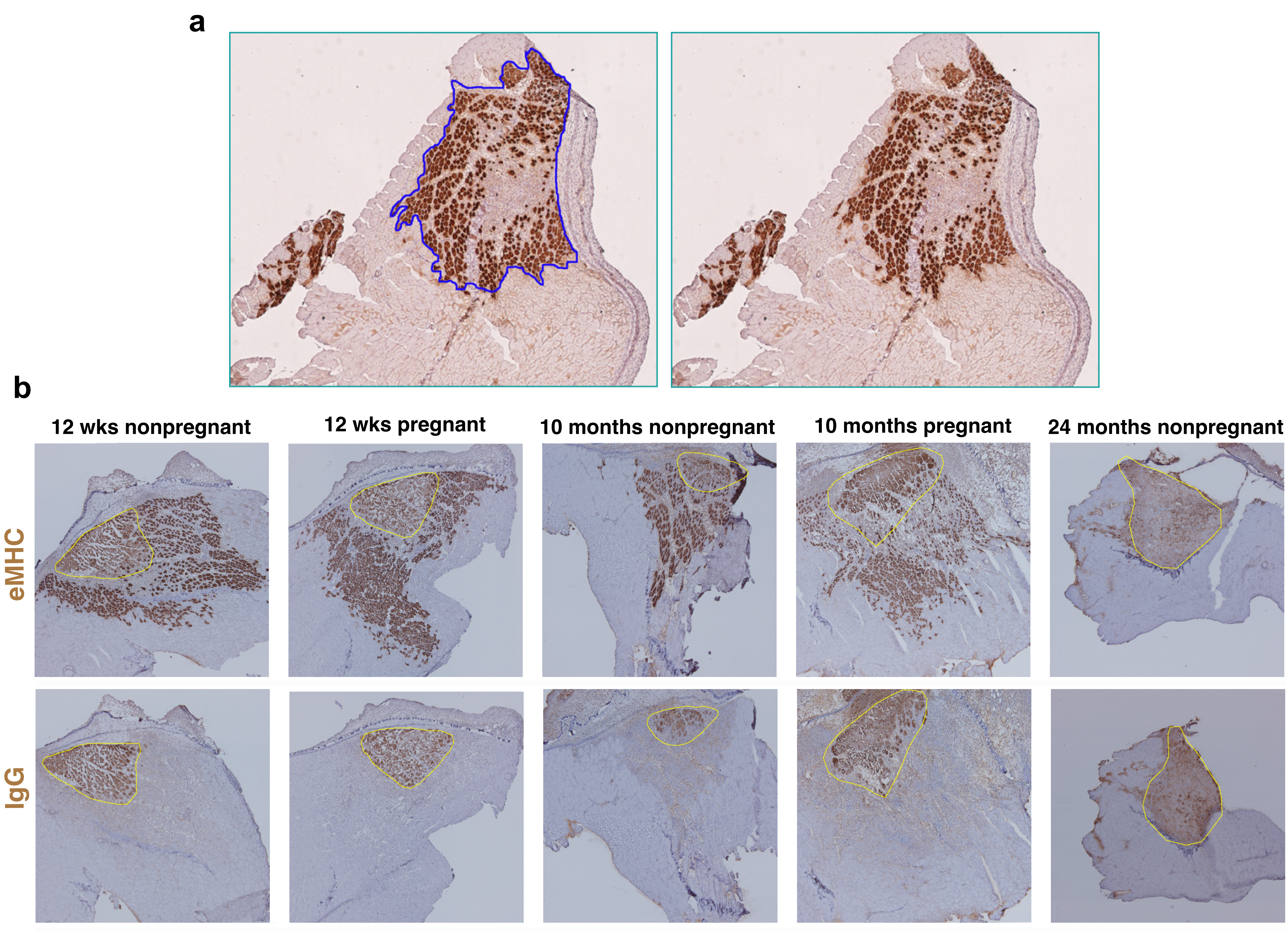
a
b
12 wks nonpregnant
12 wks pregnant
10 months nonpregnant
10 months pregnant
24 months nonpregnant
eMHC
IgG
