# Supplementary figures and images for: The rejuvenating effect of pregnancy on muscle regeneration
Source: Aging Cell. 2015 Mar 13;14(4):698–700. doi: 10.1111/acel.12286 (PMC4531083; doi:10.1111/acel.12286)

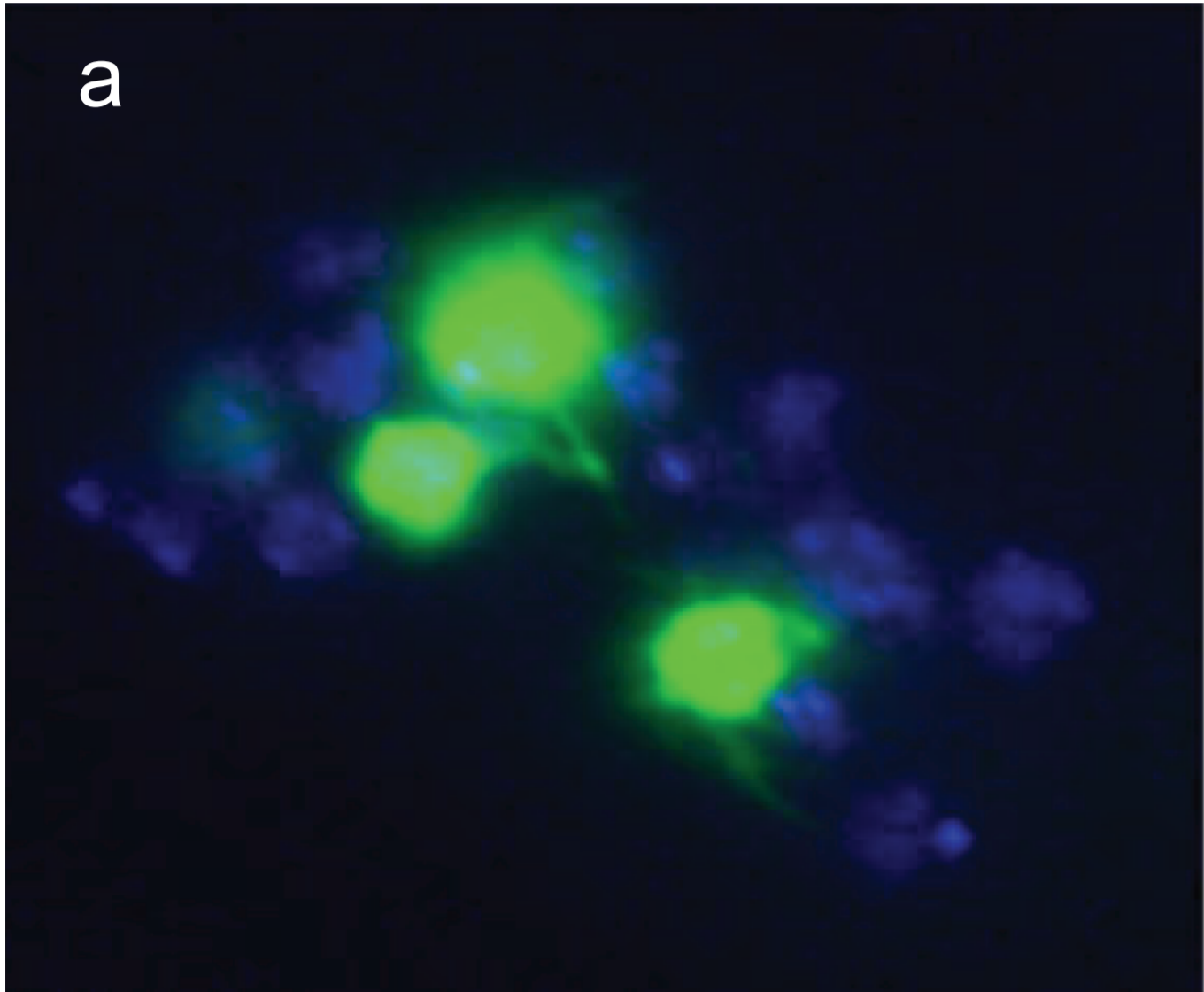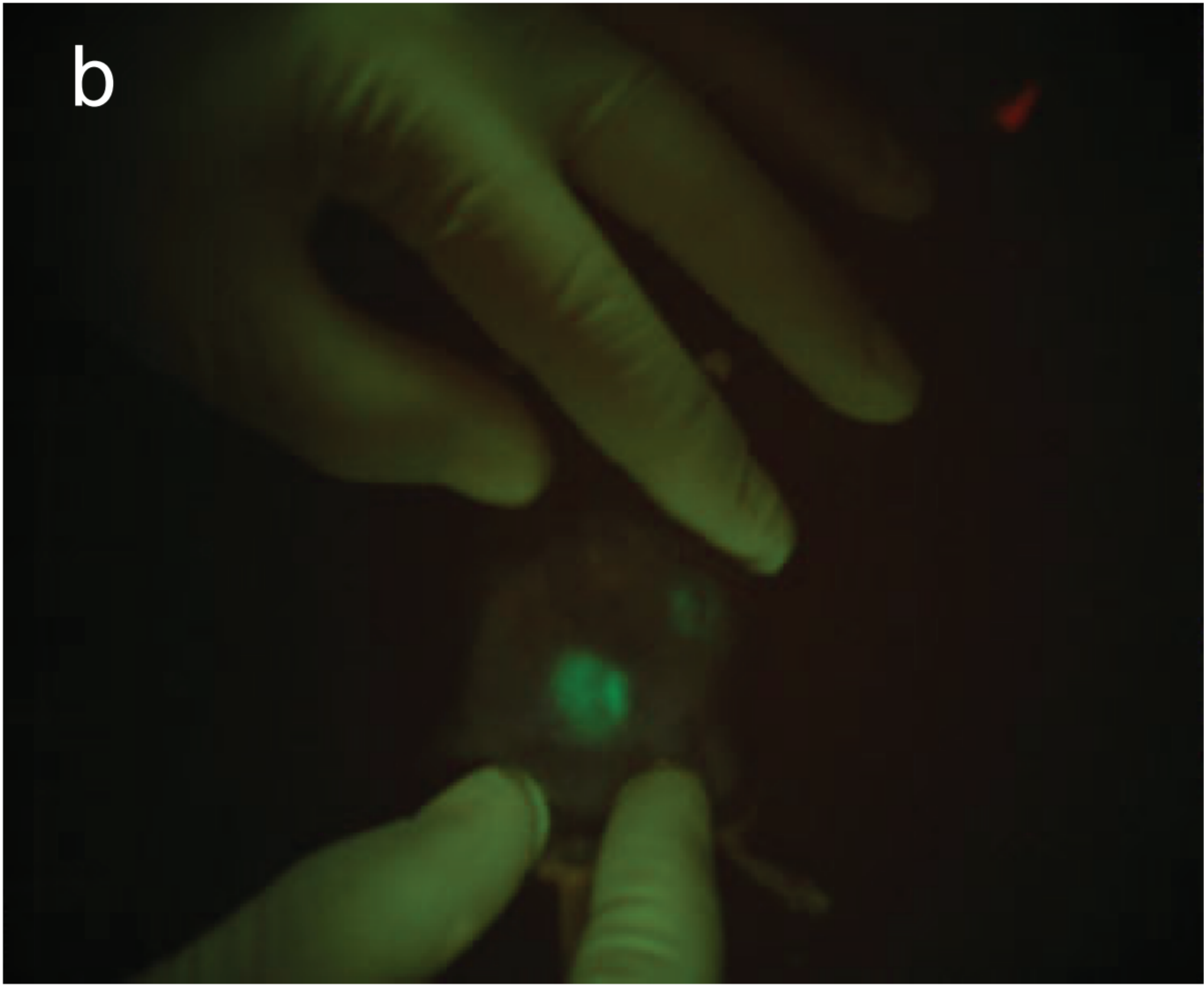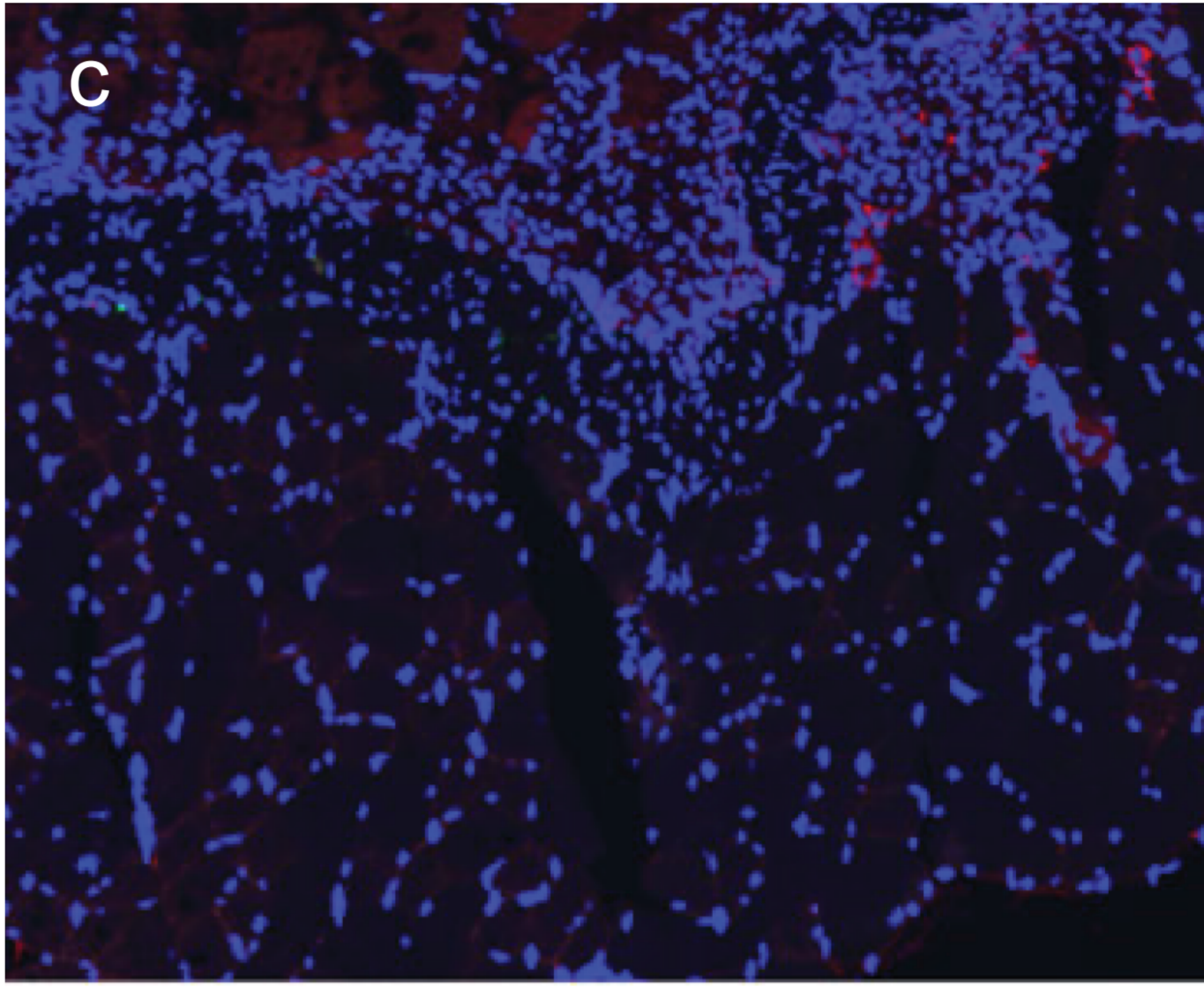

Supplement: Supplementary file 3 [file acel0014-0698-sd3.pdf]
